# LINC01087 inhibits glioma cell proliferation and migration, and increases cell apoptosis via miR-384/Bcl-2 axis

**DOI:** 10.18632/aging.203478

**Published:** 2021-08-30

**Authors:** Yao-Hui Tian, Lin-Wei Jia, Zhi-Feng Liu, Yong-Han Chen

**Affiliations:** 1 Department of Neurosurgery, Cangzhou Central Hospital, Cangzhou, Hebei Province, China

**Keywords:** LINC01087, miR-384, BCL-2, glioma, progression

## Abstract

Background: Long non-coding RNA (LncRNA) is associated with disease progression. It is reported that LINC01087 is highly expressed in cancer and participates in tumorigenesis. However, whether it regulates the development of glioma has not been studied. So, the goal of this research is to determine the role of LINC01087 in gliomas and to provide potential targets for clinical treatment.

Methods: The gene expression was detected by quantitative reverse transcriptase polymerase chain reaction (QRT-PCR) and Western blotting (WB). Cell proliferation was analyzed by CCK8 and colony formation test, and apoptosis was detected by flow cytometry. Luciferase report experiment and RNA Binding Protein Immunoprecipitation confirmed the interaction between LINC01087, miR-384 and Bcl-2. The effect of regulating LINC01087 on the growth of glioma was confirmed *in vitro*.

Results: The LINC01087 expression was up-regulated in clinical glioma samples (*n* = 35). Furthermore, LINC01087 silencing can obviously suppress the proliferation of glioma cells and induce apoptosis. Mechanically, we found that LINC01087 was the molecular sponge of miR-384. LINC01087 could inhibit the miR-384 expression and boost the Bcl-2 expression through sponge expression of miR-384. The repair of Bcl-2 effectively saved the proliferation and apoptosis of glioma cells lacking LINC01087.

Conclusion: LINC01087 is highly expressed in glioma and can participate in the growth of glioma through miR-384/Bcl-2 axis. So, it is a potential therapeutic target.

## INTRODUCTION

Glioma is the most aggressive tumor [1]. A recent study found that the incidence of intracranial tumors was 21/100,000, accounting for 2% of all cancers. Thereinto, glioma accounted for more than 50%, and the 5-year survival rate was less than 15 months [2]. Currently, the clinical treatment plan for glioma hinges on the size, location, type of tumors and age of patients. It mainly includes surgery, radiotherapy and chemotherapy and gene therapy [3–5]. Nevertheless, research has found that traditional chemotherapy patients are prone to drug resistance, thus remarkably increasing the recurrence rate during treatment. It is also the main reason for the failure of clinical treatment [6]. Up to now, the pathogenesis of glioma is still vague. Thus, it’s quite remarkable to understand and probe into glioma’s molecular mechanism and employ effective methods for diagnosis and treatment.

Long non-coding RNA (lncRNA), over 200 nucleotides long, lacks protein-coding ability [7, 8]. Early studies have discovered that lncRNA plays a certain part in the development of tumors and various diseases, especially in targeted therapy, clinical diagnosis and prognosis [9, 10]. Other studies have found that it also promotes or inhibits tumor growth and increases tumor sensitivity to chemotherapy drugs in different tumors [11, 12]. For instance, Fu et al. found that LncRNA PVT1 could accelerate tumorigenesis and glioma progression by regulating miR (microRNA)-128-3p/GREM1 axis and BMP signal pathway [13]. Another research found that TMPO-AS1, a proliferation-associated long non-coding RNA, was a latent therapeutic target for triple-negative breast cancer [14]. LINC01087, as a newly discovered lncRNA, has been studied relatively little in tumors. Currently, known references show that it is highly expressed in breast cancer, and can mediate miR/mRNA axis to take part in tumor occurrence [15]. We discovered that LINC01087 was also highly expressed in glioma through GEO (gene expression omnibus) chip analysis, and predicted that it might mediate miR-384/Bcl-2 axis to take part in the development of glioma.

Therefore, this research aims to probe into LINC01087’s expression and potential mechanism in glioma and provide potential targets for clinical treatment.

## RESULTS

### Expression of LINC01087 increases in glioma

**Figure 1:**
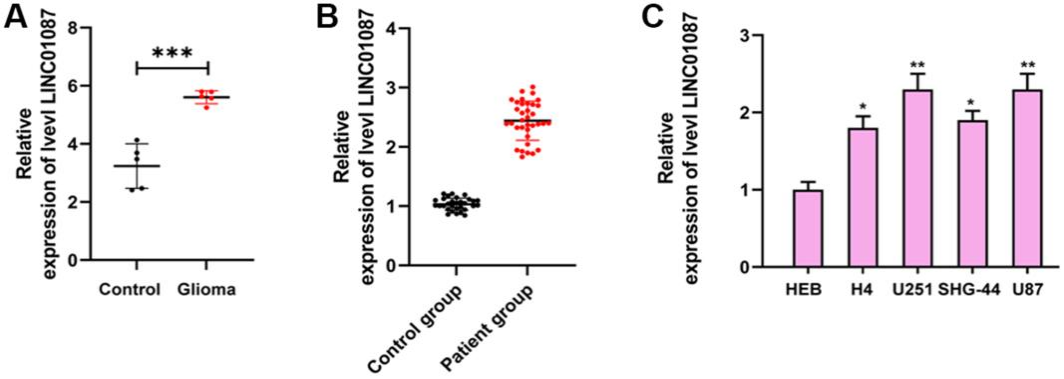
**Expression of LINC01087 in glioma.** (**A**) Relative expression of LINC01087 in GSE103229 expression profiling (^***^P < 0.001). (**B**) The relative expression of LINC01087 in tumor tissues of glioma patients was detected by qRT-PCR (^***^P < 0.001). (**C**) The relative expression of LINC01087 in glioma cells was tested by qRT-PCR (^*^means *P* < 0.05 compared with HEB cells; ^**^means *P* < 0.01 compared with HEB cells).

By analyzing the LINC01087 expression in GSE103229 expression profiling, we found that the expression in glioma increased dramatically (Figure 1A). qRT-PCR also manifested that the expression in glioma patients was obviously higher than that in the CG (Figure 1B). In addition, by detecting the expression in glioma cell lines, we also discovered that the LINC01087 relative level in U87, SHG-44, U251 and H4 cells increased markedly (Figure 1C). This suggested that it might be involved in the development of glioma.

### Knocking down LINC01087 can suppress growth of glioma and induce apoptosis

**Figure 2:**
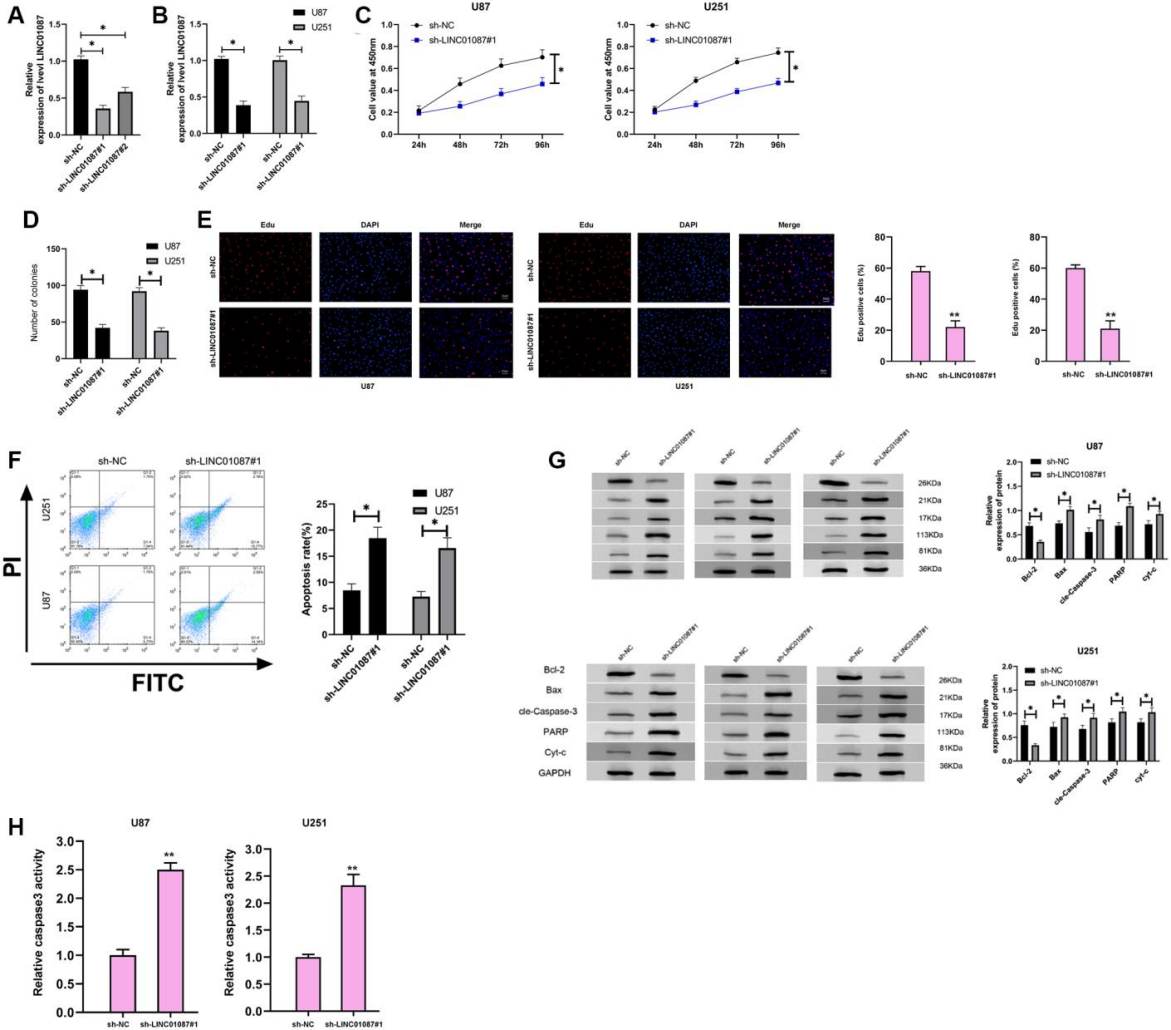
**Down-regulating LINC01087 can inhibit the proliferation of glioma cells and induce apoptosis.** (**A**) The relative expression of LINC01087 in the constructed sh-LINC01087 plasmid was tested by qRT-PCR. (**B**) The relative expression in glioma cells transfected with sh-LINC01087#1 was detected by qRT-PCR. (**C**) Viability of glioma cells transfected with sh-LINC01087#1 was measured by CCK-8 test. (**D**) Colony-formation ability changes of glioma cells transfected with sh-LINC01087#1 were tested by clone experiment. (**E**) Proliferation of glioma cells transfected with sh-LINC01087#1 was analyzed by Edu assay. (**F**) The apoptosis rate of glioma cells transfected with sh-LINC01087#1 was detected by flow cytometry. (**G**) The changes of apoptosis-related proteins in glioma cells transfected with sh-LINC01087#1 were analyzed by WB. (**H**) The relative caspase-3 activity was measured by the caspase-3 activity kit. (^*^P < 0.05).

To further understand the role of LINC01087 in glioma, we established sh-LINC01087 plasmid and transfected sh-LINC01087#1 into U87 and U251 cells (Figure 2A, 2B). Cell growth and apoptosis were tested via CCK-8, cloning experiment, flow cytometry and WB experiment. CCK-8, colony formation, and Edu assays manifested that cell viability, cloning, and proliferation were markedly suppressed after transfection of sh-LINC01087#1 (Figure 2C–2E), while flow cytometry manifested that this induced apoptosis (Figure 2F). Moreover, WB experiments showed that the expression of Bax, cle-Caspase-3, PARP and cyt-c protein after transfection was obviously higher than that of sh-NC group, while the Bcl-2 protein expression was dramatically lower (Figure 2G). Meanwhile, the depletion of LINC01087 by sh-LINC01087#1 enhanced the relative caspase-3 activity in the glioma cells (Figure 2H). It indicated that inhibiting LINC01087 could induce apoptosis and reduce glioma cell proliferation.

### There is a regulatory relationship between LINC01087 and miR-384, Bcl-2

**Figure 3:**
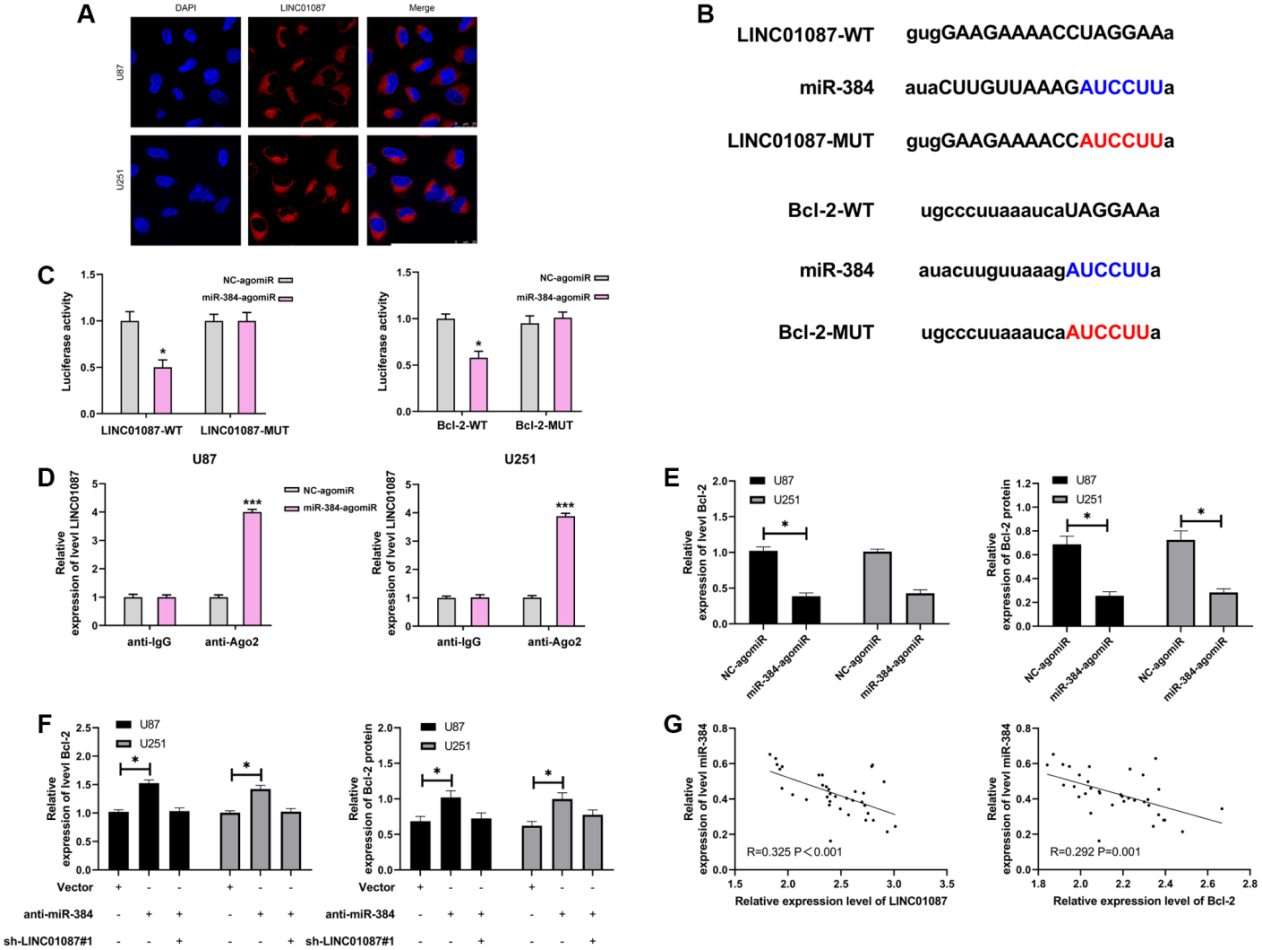
**LINC01087 regulates miR-384 to take part in the proliferation and apoptosis of glioma.** (**A**) Fluorescence *in situ* hybridization determined the distribution of LINC01087 in glioma cells. (**B**) Targeted binding and mutation sites of miR-384, LINC01087 and Bcl-2. (**C**) The effect of miR-384-agomiR on fluorescence activity of LINC01087-WT and Bcl-2-WT was tested by dual luciferase report. (**D**) miR-384 was identified in LINC01087 complex. (**E**) The relative expression changes of Bcl-2 mRNA and protein in glioma cells after transfection of miR-384-agomiR were tested by qRT-PCR and WB test. (**F**) The relative expression changes of Bcl-2 mRNA and protein in glioma cells after co-transfection of sh-LINC01087#1 and anti-miR-384 were tested by qRT-PCR and WB test. (**G**) The correlation between miR-384 and LINC01087, Bcl-2 in glioma patients was analyzed by Pearson test. (^*^P < 0.05, ^***^P < 0.001).

LncRNA has been proved to be miRNA sponge in tumor cells. To study whether LINC01087 could regulate miR, we first determined its localization in glioma cells by fluorescence *in situ* hybridization (Figure 3A). After that, we found that LINC01087 was primarily situated in the cytoplasm of glioma cells. Then, we predicted that there was a potential binding site between miR-384 and LINC01087 through starBase website. To prove this, we verified it by RIP and dual-luciferase report experiment (Figure 3B). It manifested that miR-384-agomiR could effectively inhibit the fluorescence activity of LINC01087-WT (Figure 3C), while RIP analysis found that both LINC01087 and miR-384 were enriched by anti-Ago2 in glioma cell lysate. LINC01087 could regulate miR-384 (Figure 3D), and it’s worth noting that the expression of miR-384 increased markedly after LINC01087 was knocked down. To further understand the mechanism of LINC01087/miR-384 axis, we predicted the potential mRNA downstream of miR-384. There was a targeted binding site between miR-384 and Bcl-2 through starBase and TargetScan prediction. For further verification, we conducted a dual-luciferase report. The results indicated that the fluorescence activity of Bcl-2-WT could be inhibited by miR-384-agomiR, and the Bcl-2 mRNA and protein expression in glioma cells transfected with miR-384-agomiR was obviously inhibited (Figure 3E, 3F). To determine whether Bcl-2 was regulated by LINC01087/miR-384 axis, we co-transfected sh-LINC01087#1 and anti-miR-384, respectively. In the end, we found that anti-miR-384 alone up-regulated Bcl-2 mRNA and protein expression. However, after co-transfection of sh-LINC01087#1 and anti-miR-384, there was no marked difference in Bcl-2 mRNA and protein expression compared with Vector, which was observed by qRT- PCR and Western blot analysis. Furthermore, through correlation analysis, we found that miR-384 was negatively correlated with LINC01087 and Bcl-2 expression in tumor tissues of glioma patients (Figure 3G). These studies suggested that LINC01087 could regulate miR-384/Bcl-2 axis.

### LINC01087 regulates miR-384/Bcl-2 to participate in glioma proliferation and apoptosis

**Figure 4:**
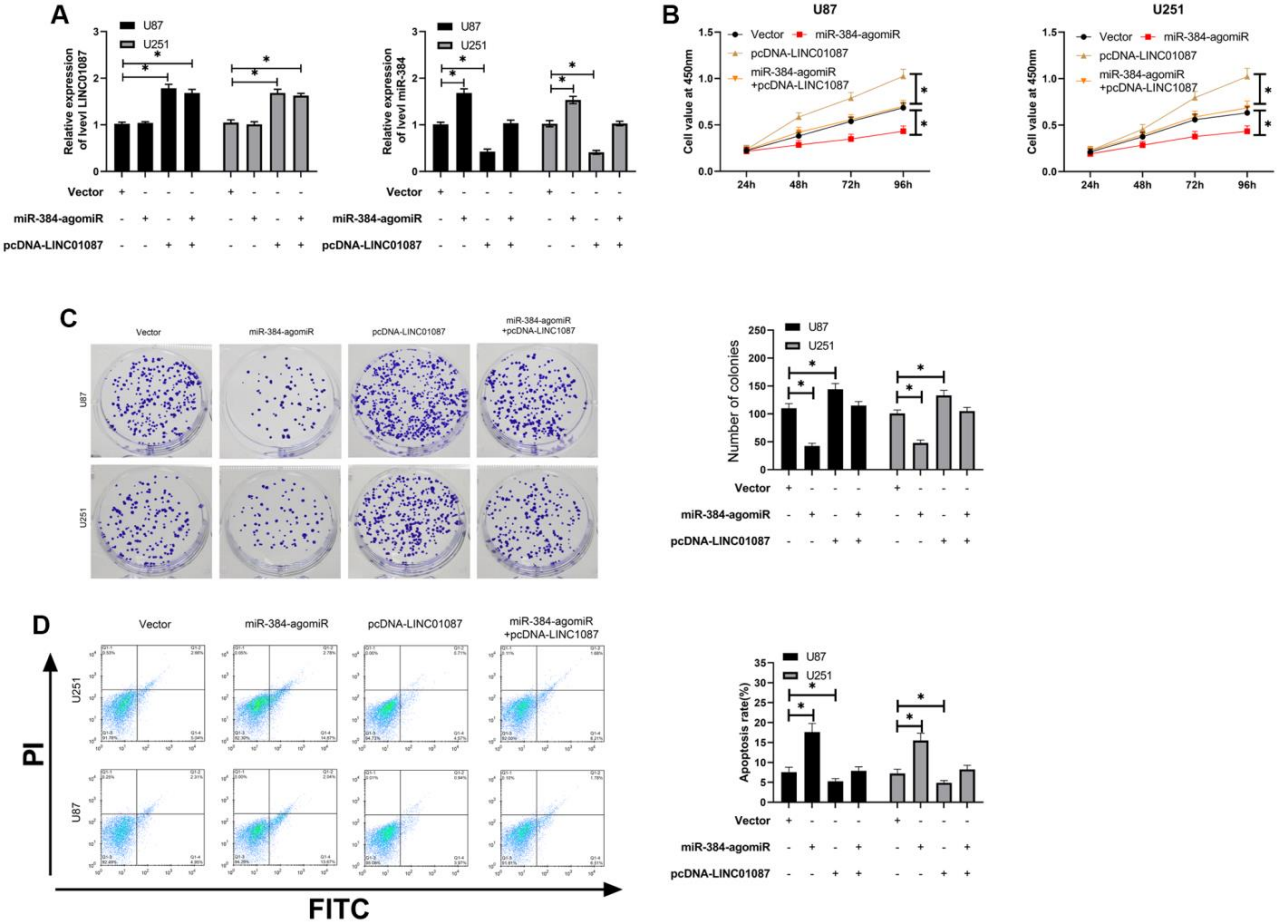
**LINC01087 regulates miR-384/Bcl-2 to take part in the proliferation and apoptosis of glioma.** (**A**) qRT-PCR detected the relative expression levels of LINC01087 and miR-384 in cells after co-transfection. (**B**) Proliferation of glioma cells after co-transfection was tested by CCK-8. (**C**) The changes of cloning of glioma cells after co-transfection were detected by cloning experiment. (**D**) The apoptosis rate of glioma cells after co-transfection was tested by flow cytometry. (^*^P < 0.05).

In the above study, we have confirmed that LINC01087 can regulate miR-384/Bcl-2 axis, but it's vague whether it is involved in glioma growth. Hence, we conducted a rescue experiment. First of all, qRT-PCR detection found that up-regulating miR-384 had no effect on LINC01087, but pcDNA-LINC01087 could inhibit the over-expression of miR-384 after transfection of miR-384-agomiR (Figure 4A). Afterward, we found that the proliferation and cloning of cells were obviously enhanced and the apoptosis rate decreased after transfection of pcDNA-LINC01087 alone, while the proliferation and cloning were obviously inhibited after transfection of miR-384-agomiR alone, which induced apoptosis (Figure 4B–4D). However, after co-transfection of pcDNA-LINC01087 and miR-384-agomiR, they were proliferated and cloned. This suggested that LINC01087 could take part in glioma growth through miR-384/Bcl-2 axis (^*^P < 0.05).

**Figure 5:**
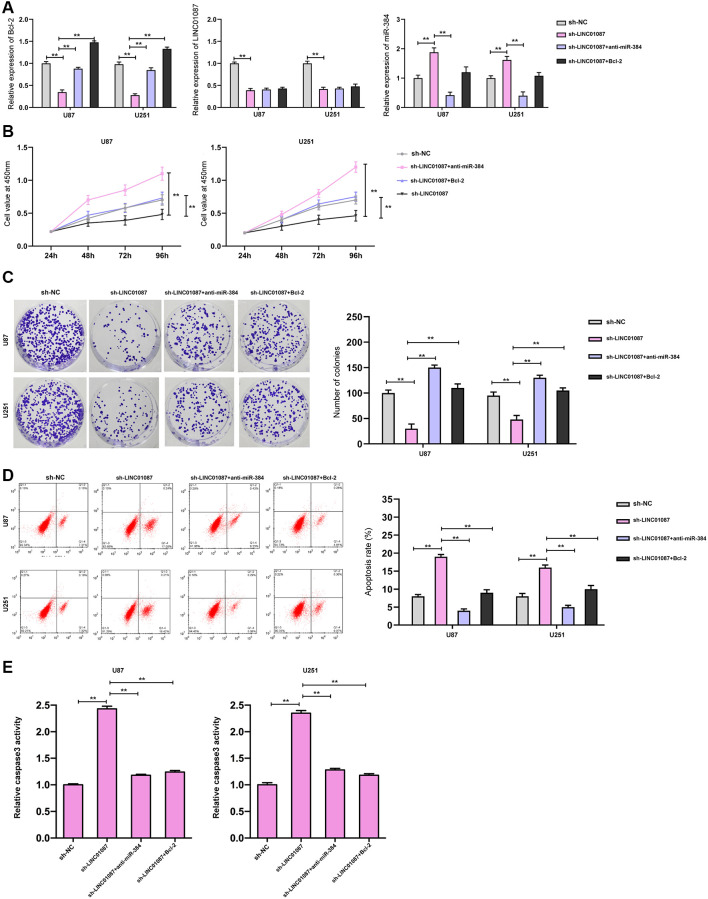
**LINC01087 regulates miR-384/Bcl-2 to take part in the proliferation and apoptosis of glioma.** (**A**) qRT-PCR detected the relative expression levels of Bcl-2, LINC01087, and miR-384 in cells after co-transfection. (**B**) Proliferation of glioma cells after co-transfection was tested by CCK-8. (**C**) The changes of cloning of glioma cells after co-transfection were detected by cloning experiment. (**D**) The apoptosis rate of glioma cells after co-transfection was tested by flow cytometry. (**E**) The relative caspase-3 activity was measured by the caspase-3 activity kit. (^*^P < 0.05, ^**^P < 0.01).

Moreover, we validated the expression of Bcl-2, LINC01087, and miR-384 in the glioma cells transfected with shLINC01087 or co-transfected with shLINC01087 and anti-miR-384 or pcDNA-Bcl-2 (Figure 5A). The cell viability and colony formation numbers were repressed and cell apoptosis and caspase-3 activity were induced by the depletion of LINC01087, while the inhibition of miR-384 or the overexpression of Bcl-2 could reverse the effect in the cells (Figure 5B–5E).

### Knocking down LINC01087 can suppress tumor growth in nude mice

**Figure 6:**
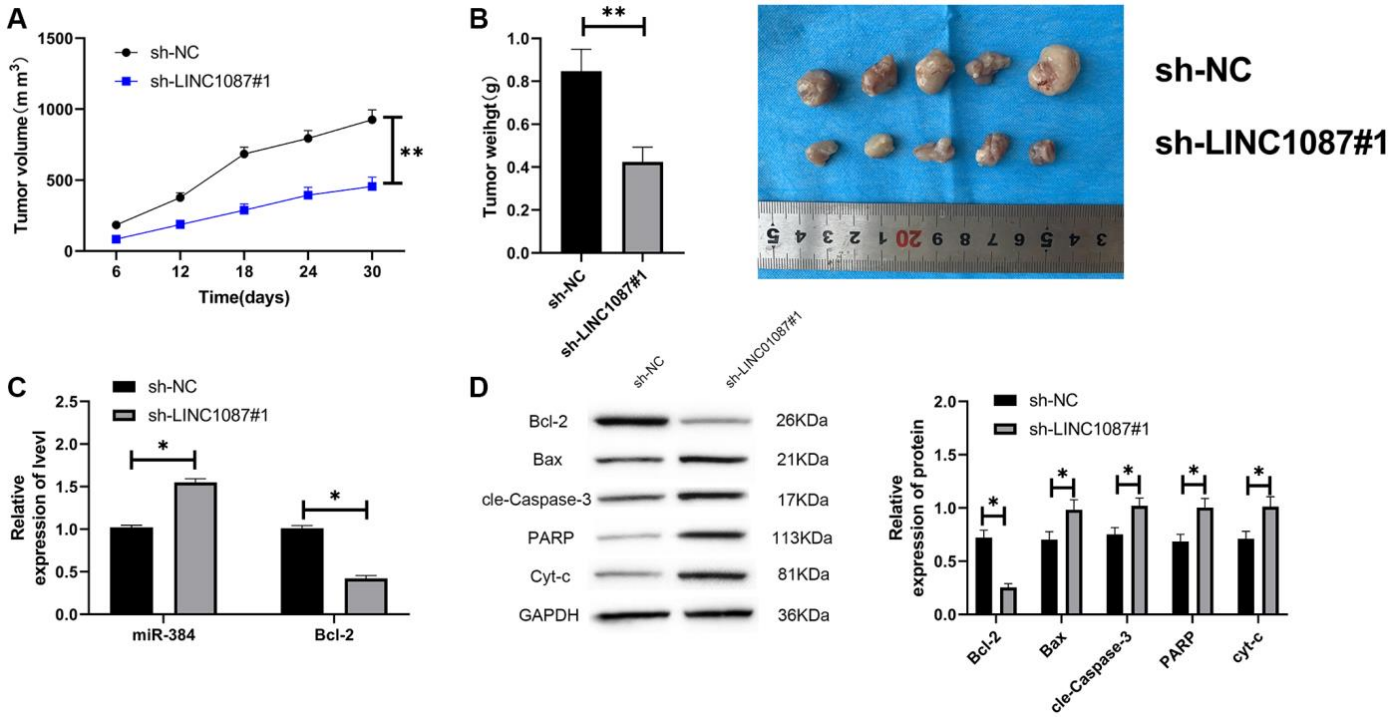
**Knocking down LINC01087 can inhibit tumor growth in nude mice.** (**A**) Changes of tumor volume in nude mice within 30 days. (**B**) Changes of tumor mass in nude mice killed 30 days later. (**C**) The relative expression of miR-384 and Bcl-2 in tumor tissues of nude mice was detected by qRT-PCR. (**D**) The expression of apoptosis-related proteins in tumor tissues of nude mice was tested by WB. (^*^P < 0.05, ^**^P < 0.01).

At the end of the study, we verified LINC01087’s effect *in vivo*. We built a xenograft tumor model and injected the glioma cells transfected with sh-LINC01087#1 into nude mice subcutaneously. We found that the tumor volume and quality of sh-LINC01087#1 group were markedly inhibited (Figure 6A, 6B), the miR-384 expression in tumor tissues increased markedly, while the Bcl-2 expression was obviously inhibited (Figure 6C). In addition, Western blot analysis showed that the expression of pro-apoptosis related proteins in tumor tissues of nude mice in sh-LINC01087#1 group increased markedly, while the Bcl-2 expression decreased dramatically (Figure 6D). It suggested that LINC01087 could suppress the growth of glioma by regulating miR-384/Bcl-2 axis.

## DISCUSSION

Recently, the morbidity of glioma has gradually increased [16]. So, finding out the pathogenesis is the key to prevent and control it. In this experiment, we found that the LINC01087 expression in glioma tissues and cells increased obviously, and it could act as miR-384 sponge and participate in the development of glioma by regulating Bcl-2.

More and more studies have shown that lncRNA acts as a competitive endogenous RNA (ceRNA) in the carcinogenesis of tumors [17]. LINC01087 is a newly discovered lncRNA located on human chromosome 2q21.1. However, the research on LINC01087 in tumors is very simple, mainly in BC [18, 19]. Our study found that the LINC01087 expression in glioma increased obviously, which suggested that it might regulate the occurrence of glioma. To further understand the mechanism of LINC01087 in glioma, we established sh-LINC01087#1 plasmid to observe its effect on glioma cell growth. However, we found that the proliferation and cloning of glioma cells decreased after knocking down LINC01087. Flow cytometry and WB experiments confirmed that the apoptosis rate of glioma cells decreased and the expression of apoptosis-related proteins increased after knocking down LINC01087. It indicated that LINC01087 might be a potential therapeutic target for glioma.

Many studies have reported that lncRNA acts as miR sponge to take part in glioma tumor development [20]. For example, lncRNA MALAT1/miR-199a/ZHX1 axis suppresses glioblastoma proliferation and progression, and lncRNA-DLEU2/miR-186-5p/PDK3 axis advances glioma cell progression [21]. It's unclear whether LINC01087 has the same mechanism in glioma. Thus, we detected its distribution in glioma cells by fluorescence *in situ* hybridization, and discovered that it mainly distributed in the cytoplasm of cells by fluorescence microscopy. It showed that Linc01087 could act as a ceRNA. Hence, we predicted through starBase that there was a potential binding site between LINC01087 and miR-384 [22, 23]. miR-384, as an early discovered miR, regulates the development of various tumors [24, 25]. For instance, Gu et al. confirmed that miR-384 was low expressed in glioma and could regulate tumor proliferation and invasion by regulating downstream target genes [26]. In this study, we found that LINC01087 could act as miR-384 sponge by dual-luciferase report and RIP experiment, and the miR-384 expression in glioma cells increased obviously after knocking down LINC01087. Bcl-2, as a complete mitochondrial outer membrane protein, can prevent the apoptosis of tumor cells and has a negative regulatory relationship with Bax. The Bcl-2 and Bax changes can be employed as a crucial basis for judging apoptosis. In this study, we predicted miR-384’s downstream target gene and discovered that there was a potential target site between Bcl-2 and miR-384. Through experiments, we discovered that miR-384-agomiR could suppress the fluorescence activity of Bcl-2, which indicated that miR-384 could regulate Bcl-2 in a targeted way. Furthermore, in our correlation analysis, we found that miR-384 was negatively correlated with Bcl-2 and LINC01087, which suggested that LINC01087/miR-384/Bcl-2 axis might exist in glioma progression.

To verify the existence of LINC01087/miR-384/Bcl-2 axis, we carried out rescue experiments. After co-transfection of pcDNA-LINC01087 and miR-384- agomiR, the proliferation, cloning and apoptosis rates of cells were obviously reversed compared with those after single transfection of pcDNA-LINC01087 and miR-384-agomiR. WB detection also found there was no remarkable difference in the apoptosis related protein expression between vector and cotransfection. What’s more, *in vitro* experiments showed that the tumor volume and mass of glioma cells injected with LINC01087 reduced markedly, and the miR-384 level increased and the Bcl-2 level decreased. This suggested that LINC01087 acted as miR-384 sponge, regulated Bcl-2 to inhibit the growth of glioma cells and induced apoptosis, which was a potential target of glioma.

Besides, we confirmed the mechanism of LINC01087 in glioma, but there are still some limitations. Firstly, the samples we detected are relatively few, and the time is short, which makes it impossible to carry out long-term follow-up and diagnosis analysis. Secondly, the samples collected this time are relatively single, and the collection of tissue samples in clinical diagnosis is generally invasive, which will cause certain damage to patients. Finally, Bcl-2 is a crucial target of cell drug resistance, and whether LINC01087/miR-384/Bcl-2 axis has the same effect in glioma drug resistance needs further verification. Hence, we hope to collect more clinical samples and carry out basic research related to drug resistance in future research to supplement our research conclusions.

To sum up, LINC01087 is highly expressed in glioma and can participate in the growth of glioma through miR-384/Bcl-2 axis. So, it's a potential therapeutic target.

## MATERIALS AND METHODS

### GEO data extraction

The GSE103229 expression profiling was searched on GEO (https://www.ncbi.nlm.nih.gov/gds/). The data were analyzed by the limma package, and the LINC01087 expression data were extracted from the GSE103229 expression profiling, and then the histogram was drawn.

### Clinical data

Thirty-five glioma patients who were treated in our hospital from January 2016 to May 2018 were selected as the research samples (25 male and 10 female patients). All of them underwent surgery. The tumor tissues of patients were collected, and the samples were sent to the pathology department of our hospital, and the glioma was diagnosed. Another 30 patients (20 males and 10 females) who underwent brain tissue resection due to craniocerebral trauma at the same time were considered as a control group (CG) (Table 1), and all the samples were stored at –80°C. All patients had not received anti-tumor treatment before. Finally, the studies involving human participants were reviewed and approved by the Cangzhou Central Hospital. All patients agreed and signed an informed consent form. This research was in line with the Declaration of Helsinki [27].

**Table 1:** Baseline data

**Factors**		**LINC01087 relative expression**		**Control group (*n* = 30)**	***P* value**
		**High expression group** **(*n* = 18)**	**Low expression group** **(*n* = 17)**		
Gender					0.753
	Male	12	13	20
	Female	6	4	10
Age					0.855
	≥45 years old	9	10	17
	<45 years old	9	7	13
Tumor size					0.505
	≥5cm	10	7	
	<5cm	8	10	
PTBE					0.471
	≥1cm	7	4	
	<1cm	11	13	
WHO stage					0.014
	I + II	6	12	
	III + IV	12	3	
IDH	Wild type	13	10		0.0036
	mutation	7	5	
ATRX	Wild type	7	9		0.1597
	mutation	9	10	
p53	Wild type	7	8		0.0577
	mutation	9	11	
1p19q	exist	6	5		0.0121
	without	11	13	

Abbreviation: PTBE: Peritumoral brain edema.

### Cell source and transfection

Human glioma cells U87, SHG-44, H4 and normal cells HEB were bought from American Type Culture Collection. Human glioma cells U251 were obtained from Procell Life Science and Technology Co., Ltd. (Wuhan, China). U87, SHG-44, U251 and H4 and were bought and then cultivated in a Dulbecco’s modified Eagle’s medium (DMEM, Gibco, NY, USA) consisting of 10% fetal bovine serum (FBS, Gibco, NY, USA). It was a humid incubator (37°C, 5%CO_2_).

Targeted LINC01087 short hairpin RNA (sh-LINC01087#1, sh-LINC01087#2) and control (sh-NC), LINC01087 over-expression plasmid (pcDNA-LINC01087) and control (pcDNA-NC), miR-384 mimics (miR-384-agomiR), inhibitor (anti-miR-384), and negative control (NC-agomiR, anti-NC) were designed and synthesized via GenePharma (Shanghai, China). Cells were transfected with Lipofectamine 3000 kit in light of the instructions.

### qRT-PCR

Total RNA was extracted from glioma cells and tissues by TRIzol reagent, and its purity and concentration were detected. The TaqMan miRNA reverse transcription kit (Thermo Fisher) was put into use, and fluorescence quantitative PCR was used for quantitative polymerase chain reaction (real-time qPCR). The reaction system and conditions referred to the kit instructions. In this experiment, GAPDH was used as lncRNA’s internal reference, and mRNA and U6 were used as miR’s internal reference. It was carried out on ABI 7500 real-time PCR system (ABI, USA), and the Gene relative expression was counted via 2^-ΔΔCT^ method [28]. Primer sequence was shown in Table 2.

**Table 2:** Primer sequence.

**Gene**	**Upstream sequence (5′-3′)**	**Downstream sequence (5′-3′)**
LINC01087	ATGCTGTATTCTATGTCCTTTACCC	CTAATGCCACAAGCCTTTTCCT
miR-384	TGAAAATGTGGACTAGAGCCAGA	CAGACACTCCAGAACAGGGC
Bcl-2	AGTGGGATGCGGGAGATGT	CGGGCTGGGAGGAGAAGA
β-actin	GTATCCTGACCCTGAAGTACC	TGAAGGTCTCAAACATGATCT
U6	CTCGCTTCGGCAGCACA	AACGCTTCACGAATTTGCGT

### CCK-8 detection

Cell proliferation changes were tested by the Cell Counting Kit-8 (CCK8, Daojin, Japan). Forty-eight hours after transfection, the cells were collected, resuspended, adjusted to 2×10^3^, and inoculated into 96-well plates. Then, 10 μL CCK8 solution was supplemented and cultivated for 2 h at 24, 48, 72 and 96 h, respectively. Subsequently, the absorbance at 450 nm was examined.

### Clonal formation detection

The clone-forming ability of glioma cells was detected by the Colony Formation Assay and 500 cells were inoculated into a 6-well plate and cultivated for 14 days. Then, the colonies were immobilized with methanol and dyed with 0.1% crystal violet. Finally, the number was counted.

### Edu assay

For EdU assay, cells were transfected with indicated plasmids and oligonucleotides and seeded in 96-well plates, followed by staining with EdU and DAPI reagent, respectively. The labelled cells were observed and photographed in a fluorescence microscope (Leica, Germany).

### Flow cytometry analysis

The apoptosis was tested by the Annexin V-PE/7-AAD (BD, Biosciences). Glioma cells were collected 48 h after transfection, digested with trypsin, centrifuged and resuspended with PBS buffer. Next, they were cleaned, fixed with precooled ethanol at 70°C for 2 h (4°C), and centrifuged. After that, they were washed with PBS buffer, rinsed twice with cold PBS, and dyed with Annexin V-PE/7AAD at indoor temperature for 15 min. The apoptotic fractions were assessed via flow cytometry (FACScan, BD Biosciences).

### Caspase-3 activity

The activities of caspase-3 were analyzed by using caspase-3 colorimetric kits (Beyotime, China) according to the manufacturers’ guidelines.

### WB detection

Total protein in transfected cells was extracted with RIPA buffer, and its concentration was examined via BCA protein analysis kit (Beyotime, Shanghai, China). The samples were separated by SDS-PAGE (Beyotime, Jinagsu, China) and transferred to PVDF membrane (Millipore, MA, USA). Afterward, the membrane was closed with 5% non-fat milk and cultivated with primary antibodies (Bcl-2 1:1000, Bax 1:1000, cle-Caspase-3 1:500, PARP 1:1000, cyt-c 1:1000) at 4°C all night long. Next, it was cultured with HRP-coupled goat anti-rabbit IgG secondary antibody (1: 2000) for 2 h. In the end, the protein was visualized under Image Quant LAS 4000 mini system.

### RIP experiment

The EZ-Magna RIP kit (Millipore, MA, USA) was applied in RNA immunoprecipitation (RIP) analysis based on the manufacturers’ protocol. The whole cell lysate was cultivated with RIP magnetic beads combined with anti-Argonaute2 antibody and normal mouse IgG (Millipore). Immunoprecipitated RNA was obtained after being incubated with proteinase K buffer. At last, the existence of binding target was verified by qRT-PCR.

### Fluorescence *in situ* hybridization

The LINC01087 probe was designed and synthesized by RiboBio (Guangzhou, China), and the sequence could be provided upon request. In view of the instructions, the probe signal was tested by FISH kit (RiboBio, Guangzhou, China). In short, GBM cells were immobilized in 4% formalin for 15 min. Cells were prehybridizated in PBS and then hybridized in hybridization solution at 37°C for 30 min. Next, the nuclei were re-stained by DAPI (4′, 6-diamidino-2-phenylindole) staining (Beyotime, China). Images were caught by a fluorescence microscope (Leica, Germany).

### Dual-luciferase reporter (DLR)

The predicted miR-384 binding sequences and their corresponding mutation sequences in LINC01087 and Bcl-2 3′UTR sequences were cloned into pmirGLO dual-luciferase vector to construct luciferase reporter vector (LINC01087-WT or -MUT and Bcl-2-3′UTR-WT or -MUT; GenePharma). HEK293T cells were inoculated into a 96-well plate and co-transfected with the vector and miR-384-agomir or its negative control. Forty-eight hours later, dual-luciferase assay was conducted. The relative luciferase activity was normalized to the luciferase activity of Renilla and counted via DLR gene assay system (Promega, WI, USA).

### Xenograft tumor models

Ten BALB/c nude mice (five weeks old) were bought from Shanghai Experimental Animal Center (China). The animal tests were performed in strict line with the regulations of the Animal Ethics Committee of our hospital and the American Guidelines for the Management and Use of Experimental Animals. U87 cells (1×10^7^) transfected with sh-LINC01087 or corresponding negative control (NC) were collected and injected subcutaneously into each side of nude mice. Thirty days later, these mice were euthanized. The weight and volume of tumors growing under the skin of nude mice were measured. The tumor volume was counted via the equation: volume = 0.5×length×width 2. The animal study was reviewed and approved by the Cangzhou Central Hospital, and it was in accordance with the National Institutes of Health guide for the care and use of Laboratory animals.

### Statistical analysis

GraphPad 7 software package was used for image rendering and data analysis. The measurement data were analyzed by K-s test, in which the normal distribution data were represented by mean ± standard deviation (Mean ± SD). The comparison between groups adopted independent sample *t*-test, and that among groups was analyzed using one-way ANOVA, marked as F. LSD-*t* test was applied in afterwards pairwise comparison, and expression among multiple time points was compared using repeated measures ANOVA, denoted by F. Bonferroni was used for back testing. Correlations between genes were analyzed using Pearson test (R = r^2^). *P* < 0.05 denoted that the differences were statistically marked.
